# An essay on ecosystem availability of *Nicotiana glauca* graham alkaloids: the honeybees case study

**DOI:** 10.1186/s12898-020-00325-3

**Published:** 2020-11-06

**Authors:** Konstantinos M. Kasiotis, Epameinondas Evergetis, Dimitrios Papachristos, Olympia Vangelatou, Spyridon Antonatos, Panagiotis Milonas, Serkos A. Haroutounian, Kyriaki Machera

**Affiliations:** 1 Laboratory of Pesticides’ Toxicology, Department of Pesticides Control and Phytopharmacy, Benaki Phytopathological Institute, 8 St. Delta str., 14561 Kifissia, Attica Greece; 2 Laboratory of Nutritional Physiology and Feeding, Agricultural University of Athens, Iera Odos 75, 11855 Athens, Greece; 3 Laboratory of Agricultural Entomology, Department of Entomology and Agricultural Zoology, Benaki Phytopathological Institute, 8 St. Delta str., 14561 Kifissia, Attica Greece; 4 Biological Control Laboratory, Department of Entomology and Agricultural Zoology, Benaki Phytopathological Institute, 8 St. Delta str., 14561 Kifissia, Attica Greece

**Keywords:** *Nicotiana glauca*, Anabasine, Nicotine, Invasive plants, Honeybees, HILIC

## Abstract

**Background:**

Invasive plant species pose a significant threat for fragile isolated ecosystems, occupying space, and consuming scarce local resources. Recently though, an additional adverse effect was recognized in the form of its secondary metabolites entering the food chain. The present study is elaborating on this subject with a specific focus on the *Nicotiana glauca* Graham (Solanaceae) alkaloids and their occurrence and food chain penetrability in Mediterranean ecosystems. For this purpose, a targeted liquid chromatography electrospray tandem mass spectrometric (LC–ESI–MS/MS) analytical method, encompassing six alkaloids and one coumarin derivative, utilizing hydrophilic interaction chromatography (HILIC) was developed and validated.

**Results:**

The method exhibited satisfactory recoveries, for all analytes, ranging from 75 to 93%, and acceptable repeatability and reproducibility. Four compounds (anabasine, anatabine, nornicotine, and scopoletin) were identified and quantified in 3 N*. glauca* flowers extracts, establishing them as potential sources of alien bio-molecules. The most abundant constituent was anabasine, determined at 3900 μg/g in the methanolic extract. These extracts were utilized as feeding treatments on *Apis mellifera* honeybees, resulting in mild toxicity documented by 16–18% mortality. A slightly increased effect was elicited by the methanolic extract containing anabasine at 20 μg/mL, where mortality approached 25%. Dead bees were screened for residues of the *N. glauca* flower extracts compounds and a significant mean concentration of anabasine was evidenced in both 10 and 20 μg/mL treatments, ranging from 51 to 92 ng/g per bee body weight. Scopoletin was also detected in trace amounts.

**Conclusions:**

The mild toxicity of the extracts in conjunction with the alkaloid and coumarin residual detection in bees, suggest that these alien bio-molecules are transferred within the food chain, suggesting a chemical invasion phenomenon, never reported before.

## Introduction

Invasive Alien Species (IAS) is a terminology recently established for the description of *taxa* presenting an aggressive expansion. According to the Convention on Biological Diversity, IAS refer to thriving populations of non-native *taxa*, which apply severe pressures on ecosystems and local biodiversity through competition, predation, and transmission of pathogens. In specific, IAS has been found to drive globally significant socio-economic, health, and ecological costs that consequent to critical risks for agriculture, forestry, and fisheries [1, 2]. Besides, IAS impacts aggravated by climate change, pollution, and human intervention in natural ecosystems emerge as the second most severe threat, after habitat loss, for ecosystems and biodiversity conservation [3].

In Europe alone, herbal IAS account to 5.789 *taxa* [4], applying pressures that have been identified as competition events, pathogens introduction and transmission [5], and pollinators decline [6, 7]. This last pressure may be explained by the consideration of herbal IAS as a significant feed resource, providing pollinators with both nectar and pollen [8–10]. The consumption of IAS nectar is a problem because of its secondary metabolites content, which has been found to present toxic effects to pollinators [11]. In the same context, and in relation to food commodities, compounds such as alkaloids are controlled under the maximum residue limits (MRLs) Regulation 396/2005 and its respective amendments [12]. MRLs are established for some of them, but not in apiculture matrices. Hence, in cases of positives’ detection, the general default MRL of 0.01 mg/kg can be applied.

Tiedeken et al. [7] elaborating further on this subject concluded that feeding on IAS might drive the exposure of native pollinators to various levels of toxic phytochemicals. Among nectar’s secondary metabolites, alkaloids consist a clear and identifiable target for the attribution of nectar toxicity [13]. Alkaloids are generally acknowledged as the cutting edge of plants defence mechanisms deterring herbivores from damaging plant tissues [14]. Mollo et al. [15], in their effort to describe the phenomenon of natural products introduction by IAS in marine ecosystems proposed the term Alien Bio-Molecules [15], in order to distinguish them by other xenobiotics of human origin, pioneering thus the study of invasive phytochemicals ecological implications.

**Fig. 1:**
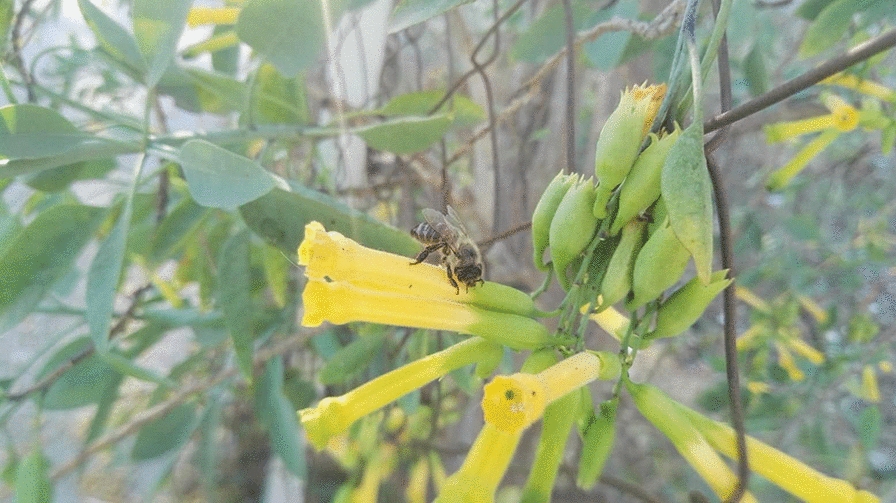
A honeybee foraging a *Nicotiana glauca* flower (photo taken by our group in Greece)

The present study is aiming to ameliorate these previous research results on the adverse effects of invasive molecules, through the investigation of their ecosystem availability. Consequently, *Nicotiana glauca* Graham (*N. glauca*, Solanaceae), whose nectar contains nicotine like alkaloids, was selected as a focal point. *N. glauca* is a fast-growing shrub or small tree native to South America that has been introduced in North America, Europe, and Asia, consisting thus an IAS of global expansion [16]. Although hummingbirds pollinate *N. glauca* in its native range [17], bees and other insects have been observed to visit its flowers and impale the base of the corolla to access its nectar (Fig. 1).

While the study of *N. glauca* flowers alkaloid content is aiming to delineate the availability of invasive molecules to indigenous pollinators communities, the use of honeybees (*Apis melifera)* fed by flowers extracts, standardised on the prevailing alkaloid concentration, aspires to investigate any potential adverse effect on bees survival after exposure. To elucidate this plant–insect interaction framework, an analytical method was developed, validated and applied to investigate alkaloids levels in plant extracts and honeybees, devoting specific focus on the *N. glauca* alkaloid content and its fate upon consumption by honeybees.

To our knowledge, the presented herein results comprise the first in depth exploration of the *N. glauca* reproductive organs in respect with their alkaloid content, even though the study of *N. glauca* alkaloid content goes back almost 75 years [18] and quite early managed to define anabasine as the prevailing pyridine alkaloid [19]. The Baldwin and Ohnmeiss [20] study provided a significant conclusion, indicating that foraging may induce alkaloid content of *N. glauca* [20], a result soon confirmed by other researchers [21]. In 2004, Tadmor-Melamed et al. presented the alkaloid content of *N. glauca* nectar and discussed on the ecological implications regarding plant’s pollinators. Kaczorowski et al. [22] indicated that foraging impacts on *N. glauca* are not restricted to morphological aspects but also may regulate the alkaloid content of nectar, promoting the biosynthesis of anabasine against nicotine. Another element of alkaloid content and foraging interaction was reported soon after by Aizenberg-Gershtein et al. [23], which correlated the foragers’ preferences with the differentiated floral microbiome as a result of alterations in alkaloid content. The final conclusion of Kaczorowski and Markman [24] that plant secondary metabolites ingestion may lead to reduced foraging performance, which in turn could significantly affect an organism’s foraging efficiency, drove our consequent experimentation aiming to delineate the alkaloids fate and impacts on honeybees that forage on *N. glauca* flowers.

Last but not least, from the alkaloids reported in *N. glauca*, and to the best of our knowledge, only for nicotine an acute contact LD_50_ > 500 μg/g bee body weight (bw), is established, which classifies nicotine as a moderate toxicant for bees [25]. Therefore, any additional data that associate alkaloids consumption to honeybees effects is of high significance, and to an extent is provided in the herein presented work.

## Materials and methods

### Materials

Herbal material consisted of *Nicotiana glauca* Graham flowers –including calyx–, which were collected from Paros island, Greece (37°07′01″ N, 25°14′08″ E), on August 9, 2015. Herbal material was dried naturally and weighted to 67 g. The dried herbal material was finely grinded and subjected to successive extraction with n-hexane, dichloromethane, and methanol. In specific each extract was obtained by the application of 1.5 L of solvent divided into three repetitions (0.5 L each) of three days duration each. The extracts were condensed through vacuum and heat-assisted evaporation (Büchi Rotavapor R-210 equipped with Büchi vacuum pump V-700, Vacuum controller V-850, and Julabo F12 cooling unit), and subsequently subjected to freeze-drying (Scientz-18 N, Freeze dryer). The yield of each solvent’s extract (in g per 100 g of dried flowers) were as follows: (a) hexane, 4.2 (b) dichloromethane, 1.8, and (c) MeOH, 12.2.

A beehive of *Apis mellifera* ssp. cecropia, a *taxon* originating in southern Greece, was used as pollinator for the study. The apiary consisted from 25 standard Langstroth hives with one hive-body containing 10 frames. The hives were not subjected in chemicals treatment for the control of pests and diseases. The bees were recruited from two brood frames of a single hive; young worker bees (3–13 days old “nurse bees”) were captured from the hive, put into a plastic box and transferred to the laboratory. In addition, dead bees were obtained from the reservoir of routine honeybee samples that Benaki Phytopathological Institute receives for pesticide residue analysis and assessment of Varroa and Nosema infestation and were utilized as control samples. These samples were devoid of pesticide residues and the herein targeted compounds.

The following chemical standards and reagents were used in the course of our study: (-)-cotinine, (R,S)-anatabine, and myosmine purchased from Santa Cruz Biotechnology, Inc., ( ±)-anabasine and scopoletin from Acros Organics, ( ±)-nicotine from Cayman Chemicals, and nornicotine from Sigma Aldrich (purity for all standards was above 90%). Acetonitrile LC–MS grade and extra pure LC–MS water was obtained from Merck (Darmstadt, Germany), magnesium sulphate (MgSO_4_) was purchased from Scharlab S.L. (Barcelona, Spain), sodium acetate from Panreac (Barcelona, Spain), primary secondary amine (PSA) from Interchim (Montluçon France), and octyldecylsilane (C18) endcapped from Macherey Nagel (Düren, Germany). Dichloromethane and hexane (pro analysis) were obtained from Fisher Scientific (Pittsburgh, USA).

### Methods

#### Bioassay

Bees feeding bioassay was conducted in groups of 40–50 individuals, using three replicates per treatment (see treatments below). The groups of 40–50 bees were placed in wire-screened plastic holding-cages (30 × 30 × 30cm) and provided with 50% (w/v) sucrose solution and water for 24 h in order to acclimatize to laboratory conditions. Bees were deprived of food for 5 h, before the administration of *N. glauca* extracts. Then, the appropriate solutions (solutions are presented below) of *N. glauca* extracts were prepared on with sucrose and water, poured into small plates (10 cm in diameter), and provided to bees.One plate containing 20 mL of the appropriate solution of *N. glauca* extracts was placed into each cage except the control were bees fed with 20 mL 50% (w/v) sucrose solution. Treated food remained in the cages for 24 h. Then, all treated plates were replaced by new plates that contained only sucrose solutions. For each treatment, three replicates of 40–50 bees were used.

#### Feeding solutions

After determining the composition of each dry extract, three stock solutions were prepared through the addition of a 50% (w/v) sucrose solution (in water) in each dry extract (devoid of organic solvent residues) benefiting from the high-water solubility of anabasine [26] and anatabine [27], and the moderate solubility of nornicotine [28], and scopoletin [29]. Then, by appropriate dilution with the addition of 50% (w/v) sucrose solution in for each feeding solution to present a constant concentration of 10 μg/mL of anabasine. The rationale behind the selection of this concentration stems from a pertinent study on the feeding of foraging honeybees to secondary molecules that mimic floral nectar [30]. In this study, bees were dissuaded by such concentration of anabasine.

In addition to these three feeding solutions (see Additional file 1: Table S1 for components’ concentration in the extracts), another methanolic solution standardised to 20 μg/mL of anabasine (methanol extract 20 μg/mL) was prepared also accompanied by the control feeding solution containing only sucrose (50%, w/v). The feeding solutions were administered in a constant dose of 20 mL per cage, which remained in the cages for 24 h and then were replaced by 50% (w/v) sucrose solutions. Honeybees’ mortality was assessed in 0, 4 and 8 days post-treatment.

#### Liquid chromatography—tandem mass spectrometry

An Agilent Technologies 6410 Triple Quad LC/MS system was used, equipped with a Nitrogen Generator (NitroFlowLab). Optimum LC separation condition, using HILIC conditions described in the literature [31] were embraced with the flow rate modified to 0.4 mL/min, working under isocratic conditions. Thus, the mobile phase was composed of a mixture of acetonitrile and water (85:15, v/v) containing 5 mmol/L ammonium acetate (pH 5). The sample injection volume was 10 μL. Separation occurred on a HILIC column (Agilent, Zorbax HILIC Plus, Narrow Bore RR, 2.1 × 100 mm, 3.5 μm).

Reversed phase (RP) separation was achieved after injecting 10 μL of sample on a RP column (ZORBAX Eclipse XDB-C18 Agilent, 2.1 × 150 mm, 3.5 μm) using a gradient system consisting of (A) Water with 5 mM ammonium formate, 0.1% formic acid, and (B) Methanol with 5 mM ammonium formate, 0.1% formic acid. The flow rate was set at 0.3 mL min^−1^ and the column gradient program consisted of: linear ramping from 0 to 100% B within 10, 10–15 min 100% B. Then gradient system returned from 15–20 min, to initial conditions (100% A), where it stayed for additional 5 min to equilibrate. The mass spectrometer was operated in Multiple Reaction Monitoring (MRM) mode with positive Electron Spray Ionization, and both quadrupoles were set at unit mass resolution. Nitrogen was used as nebulizer and collision gas. The software used for instrument control was Agilent Mass Hunter data acquisition Triple Quad B.01.04 software and for data processing Agilent MassHunter Workstation Qualitative Analysis B.01.04 software.

### Solutions and sample preparation

Stock solutions of each analytical standard were prepared at 1000 μg/mL in methanol. Subsequently, an intermediate stock solution was prepared at 10 μg/mL containing all analytes. The latter was used to prepare the working solutions used for calibration purposes. This approach was incorporated for the spiking of both plant and honeybees’ extracts. All working solutions were prepared on a daily basis, while stock solutions were kept stored at −18 °C.

Concerning the 3 dry herbal extracts, they were dissolved in methanol to provide the respective stock solutions. Consequently, each stock solution was diluted with methanol to afford an extract concentration of 100 ppm that was filtered (PTFE, 45) and then subjected to LC–ESI–MS/MS analysis.

The honeybees’ sample preparation was based on a modified “quick, easy, cheap, effective, rugged, and safe” (QuEChERS) protocol [32]. More specifically, 1 g of honeybees (approximately 10–15 dead individuals) was mixed with water (2 mL) using a glass rod, and acetonitrile was added (7 mL). The mixture was homogenized for 4 min (Ultra Turax homogenizer, 20,000 rpm) and then transferred to a falcon tube containing MgSO_4_ (1 g), and sodium acetate (0.2 g). The resulting mixture was shaken for 1 min, and vortex-mixed for 1 min. After centrifugation at 4000 rpm and 10 °C for 5 min, the upper organic phase was transferred to a separate falcon tube containing MgSO_4_ (500 mg), PSA (50 mg), and C18 endcapped (25 mg). After shaking, and vortex-mixing for total 2 min, the supernatant solution was decanted and evaporated to dryness using a nitrogen stream. The dry extract was reconstituted with acetonitrile (1 mL), filtered, and injected to the LC–ESI–MS/MS system.

### Validation methodology

The proposed method’s validation was structured upon the guidelines set by the International Conference on Harmonization [33], elaborated by later approach, pertinent to chemical measurements in natural products research [34]. As validation parameters were set:

#### Precision (intra-day, inter-day)

The precision of the chromatographic method was expressed as the relative standard deviation, RSD % of the repeatability (intra-day) and intermediate precision (inter-day) analyses (n = 3) over the three days studied, after injection of quality control samples, fortified at 200 ng/g with appropriate volume of the analytes mix solution. Intra-day, and inter-day precision were considered acceptable when RSD% were < 20%.

#### Accuracy-trueness (recovery)

Standard addition was used for the recovery study concerning the *N. glauca* flower extracts, and conducted at three concentration levels (50, 200, and 1000 ng/g). *N. glauca* plant extracts spiked with the analytes mix solution at the same concentration levels on the same day or within three different days were extracted to determine repeatability (n = 3) and reproducibility (n = 3), respectively.

#### Linearity

Linearity was acceptable when regression coefficient was higher than 0.99, and residuals were less than 20%.

#### Detection and quantitation limit (LOD and LOQ respectively)

LOD and LOQs determinations were based on the standard deviation of the response and the slope, following Eqs. 1 and 2. In regard with the honeybees’ analytical method validation, the only differentiation was the LOQ definition that was defined as the lowest fortification level with acceptable precision and accuracy.

1 $$LOD=\frac{3.3\sigma }{S}$$

Equation 1: Limit of Detection calculation algorithm

2 $$LOQ=\frac{10\sigma }{S}$$

Equation 2: Limit of Quantification calculation algorithm.

σ = standard deviation of the response,

S = slope of the calibration curve.

The calibration curves (in solvent and matrix extract) were determined using the dilute standard solution of the mixture of analytes investigated in the proposed analytical method. Calibration curves varied from 10 to 1000 ng/mL, containing seven calibration points (10, 50, 100, 200, 300, 500, 1000). To estimate if the matrix, affects the peak area of the analytes of the method significantly, therefore sensitivity, the slopes of the calibration lines obtained for plant and honeybees extract after standard addition (b_matrix_), and the solvent (b_solvent_) were divided to determine the matrix factor and the % matrix effect (ME) was calculated as indicated in Eq. 3.

3 $$\mathrm{\%ME}=\left(1-\frac{{\mathrm{b}}_{\mathrm{matrix}}}{{\mathrm{b}}_{\mathrm{solvent}}}\right)\times 100$$

Equation 3: Matrix effect calculation algorithm.

### Statistical analyses

To analyze the differences of *N. glauca* extracts on the bee mortality among the different observation days, repeated measures ANOVA (RM-ANOVA) were performed. The percentage mortalities were transformed to arcsine square-root values prior to analysis in order to satisfy assumptions of the parametric analysis. When RM-ANOVA indicated a significant difference among treatments, Bonferroni test was used to identify the differences between treatments and control. Analyses were performed using the statistical package SPSS 22.0 (SPSS Inc., Chicago, IL, U.S.A.).

## Results

### Analytical method validation

For the analytical methods validation, the dried organic extracts were fortified with known amount of the mix solution of the alkaloids and scopoletin. The outcomes of validation study, presented for the methanolic extract in Table 1, showed that all analytical figures of merit were acceptable. Recoveries, for all analytes, for the three studied concentration levels varied from 75 to 93%, with satisfactory RSD% values (≤ 15%). Similar values, (Additional file 1: Tables S2, S3), were obtained for the other two extracts and in the same magnitude. Repeatability (intra-day precision) and inter-day precision were acceptable as demonstrated by the RSD% values that were below 10.5%. More specifically, recoveries for all compounds varied from 74 to 100%, in the three concentration levels studied, with RSD% values < 11%. LODs for the plant extracts were calculated as described above and varied from 10 to 26 ng/g extract, while LOQs fluctuated from 30 to 79 ng/g extract. Concerning honeybees an LOQ (for all analytes) was established at 40 ng/g bee bw. The latter was fit for the purpose of the study and was supported by acceptable precision and accuracy, as indicated in Additional file 1: Table S4.

**Table 1:** Analytical method validation characteristics for the *Nicotiana glauca* MeOH extract

Compound	Regression equation*	Regression coefficient (R^2^)	LOD (ng/g)	LOQ (ng/g)	ME (%)	Recovery ± RSD % (n = 3)			Intra-d-precision (RSD % n = 3)	Inter-d-precision (RSD % n = 3)
						50 ng/g	200 ng/g	1000 ng/g	200 ng/g	200 ng/g
Anabasine	y = 6670049.8x − 37750.2	0.9970	19	58	2.9	81 ± 7	87 ± 15	83 ± 8	3.8	5.1
Nicotine	y = 1451011x + 14365.6	0.9997	26	79	1.9	78 ± 10	83 ± 14	93 ± 9	1.7	6.3
Anatabine	y = 4835017.3 + 251.2	0.9995	13	39	0.7	91 ± 15	87 ± 9	85 ± 12	4.7	6.2
Nornicotine	y = 41448304x − 299690.9	0.9997	10	30	5.8	76 ± 8	82 ± 14	87 ± 10	2.5	4.3
Myosmine	y = 2387692.2x − 9948.8	0.9947	11	34	4.2	80 ± 8	76 ± 7	82 ± 6	6.5	7.3
Scopoletin	y = 1839928.8x − 3670.4	0.9999	24	72	−1.9	85 ± 10	91 ± 10	90 ± 12	1.7	6.1
Cotinine	y = 12933154x − 44736.5	0.9979	11.3	33.4	5.7	75 ± 5	80 ± 8	94 ± 12	3.8	3.2

*Residuals for all concentration levels were below 16%

One often unanticipated etiology of low quality analytical results is the matrix interference. Even though the MS/MS technique is considered less susceptible than other analytical techniques and detectors (such as simple MS, or UV detector), still matrix effect is vastly reported. In this context, the assessment of matrix effects demonstrated a slight enhancement of the signal of the analytes, except for scopoletin (slight suppression), overall being characterized as not significant. Non-significant was also the ME for all analytes using the QuEChERS extraction of chemicals from bees (see ME values in Additional file 1: Table S2, and a respective blank chromatogram, Additional file 1: Figure S1). Separation of analytes under RP chromatography conditions was not successful especially for anatabine and nicotine. In particular, the resolution of their isomers could not be evidenced under such conditions (Additional file 1: Figure S2). Even though the MS/MS environment does not require substantial resolution among analytes in multianalytes methods, it was decided to enhance the separation of the compounds and their isomers, optimizing the peak shape. The latter was accomplished by utilizing hydrophilic interaction chromatography conditions [35].

### Herbal extracts

The qualitative analyses of the three *N. glauca* extracts included as target molecules the alkaloids anabasine, anatabine, nicotine, nornicotine, cotinine, myosmine, and the coumarin scopoletin (for MRM transitions and retention times see Additional file 1: Figure S3A–G). The results presented in Table 2, indicated as more diverse the methanolic extract, which contained anabasine, anatabine, nornicotine and scopoletin, while dichloromethane extract proved to contain only anabasine and anatabine, and hexane extract only anabasine. The quantitative analysis that was performed in the three *N. glauca* extracts delineated the concentrations of anabasine, anatabine, scopoletin and nornicotine. The methanolic extract exhibited the highest concentrations of all constituents, with prevailing compound anabasine (for respective chromatogram see Additional file 1: Figure S4), and lower levels of anatabine, scopoletin, and nornicotine. Dichloromethane and hexane extracts presented a much lower and descending concentration of anabasine, respectively. Nornicotine is a demethylation product of nicotine that is reported to occur in Nicotiana species [36], and was evidenced in the methanolic extract. Similarly, scopoletin, which has been reported as an active constituent of *N. glauca* [37] was quantified as well. On the other hand, nicotine, myosmine, and cotinine were not detected in any of the extracts.

**Table 2:** *Nicotiana glauca* flower extracts’ bioactive components concentration (in μg/g of dry extract material; n = 3)

Extract	Compounds					
	Anabasine	Anatabine		Scopoletin	Nornicotine	
Hexane	96.7 ± 8.5	nd		nd	nd	
Dichloromethane	770 ± 32	9.0 ± 0.8		nd	nd	
Methanol	3900 ± 156	38 ± 4.2		41 ± 3.7	11 ± 1.5	

*nd* non-detected

### Feeding bioassay

**Fig. 2:**
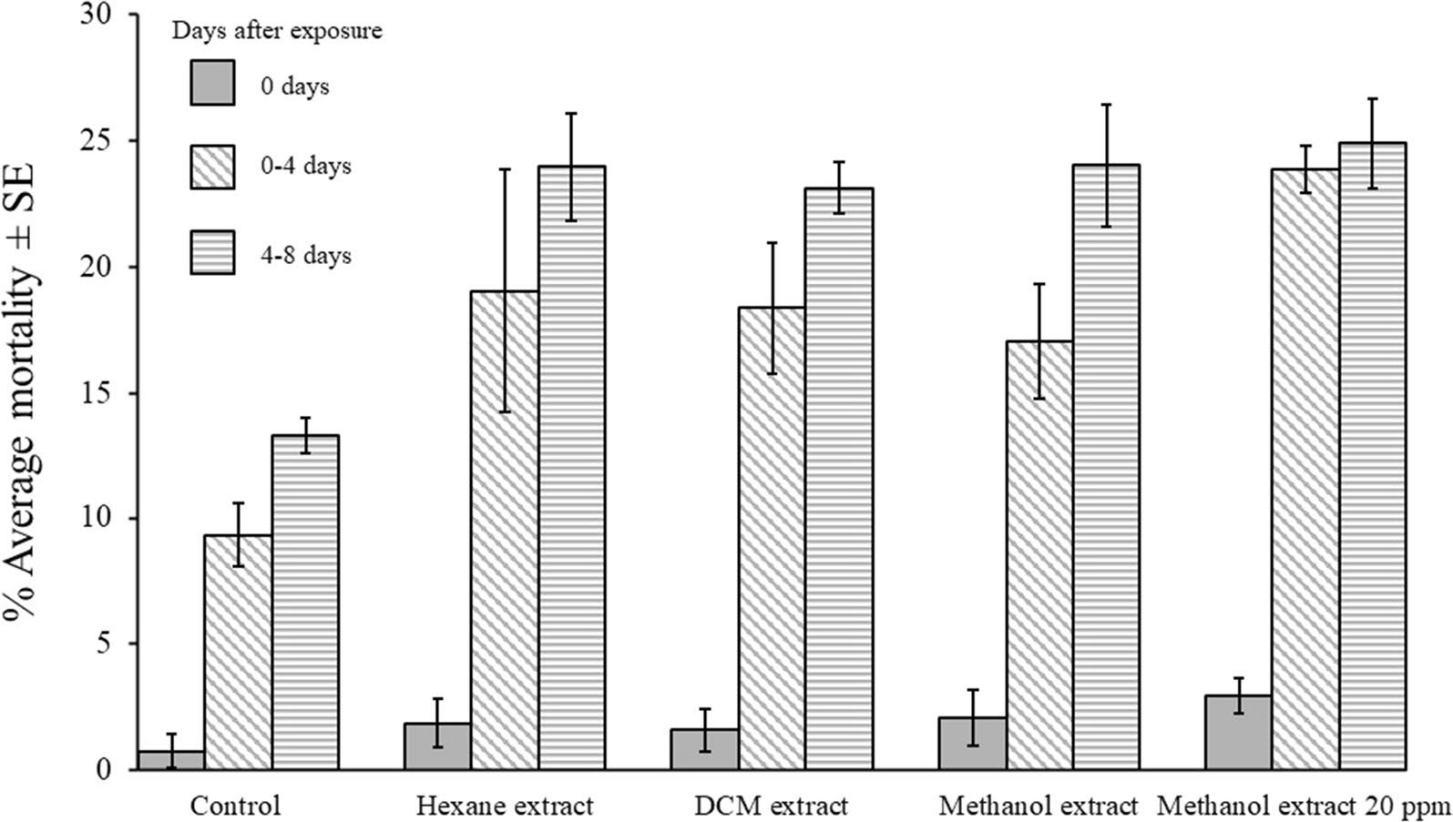
Average mortality of *Apis mellifera* young workers at 0, 4, and 8 days after feeding for 24h on *N. glauca* extracts

The feeding experiment provided two significant sets of results. The first regards the observed bees’ mortality rates, which is presented in Fig. 2; the second set consists of the records of alkaloids quantities recorded in the dead bees of the feeding bioassay, which are presented in Table 3. Control honeybees’ mortality until the 4th day did not exceed the threshold of 10% set by the Organization for Economic Co-operation and Development (OECD) in the guidelines for honeybees’ acute oral toxicity test [38].

**Table 3:** Honeybees’ daily estimated exposure to alkaloids of *Nicotiana glauca* feeding solutions (in ng per bee) and mean detected residues in dead bees (in ng per bee; n = 3)

Feeding solution	Anabasine		Anatabine		Nornicotine		Scopoletin		
	Exposure	Residue	Exposure	Residue	Exposure	Residue		Exposure	Residue
Hexane	1040	4.3 ± 0.19	nd	nd	nd	nd		nd	nd
Dichloromethane	1040	4.7 ± 0.3	12	nd	nd	nd		nd	nd
Methanol	1040	5.1 ± 0.27	10	nd	3	nd		11	< LOQ
Methanol extract 20 μg/mL	2080	9.2 ± 0.52	20	nd	6	nd		22	< LOQ

*nd* non-detected, *LOQ* 4 ng per bee or 40 ng/g _bee bw_

All *N. glauca* extracts had a significant effect on bees’ mortality compared to control (Bonferroni test: p < 0.05), presenting similar mortality rates between 16 and 18%, with the exception of methanol extract’s feeding solution containing anabasine at 20 μg/mL, which presented an augmented mortality rate by 23% in the 4th day. Both feeding treatment and post treatment period had a significant effect on bees mortality F = 21.08; df = 4, 10; p < 0.001); F = 104.08; df = 8, 20; p < 0.001). No significant interaction between the two factors was found (F = 0.27; df = 8, 20; p = 0.97).

### Residues in bees

Dead bees were analyzed for the presence of the four constituents detected in the respective extracts (see Table 3 for results). Anabasine was detected in dead bees from all the feeding solutions, confirming its uptake from the plant extracts. In specific, anabasine was found on an average concentration of 9.2 ng per bee (equivalent to 92 ng/g _bee bw_), 5.1 ng per bee, 4.7 ng per bee, and 4.3 ng per bee in methanol extract 20 μg/mL, methanol, dichloromethane and hexane extract 10 μg/mL respectively (an indicative chromatogram is presented in Additional file 1: Figure S5). Scopoletin traces were detected in dead bees from feeding solutions containing it. These residual invasive molecules quantities originated exclusively from the feeding solutions content.

### Estimates on bees daily anabasine exposure

To facilitate subsequent discussion, each *N. glauca* flower produces in Greece an average of 23.5 μL of nectar with sucrose mean concentration of 25.2% [17]. Taking into account that the minimum daily need of an adult bee for sucrose is 4000 μg [39, 40] it is estimated that each bee will consume approximately 16 μL of nectar (see Eq. 4) if all sucrose daily needs is to be covered from *N. glauca* nectar. Considering the significant fluctuation of anabasine concentration in *N. glauca* nectar ranging from 0.39 μg/mL [22], to 5 μg/mL [16] a single honeybee may be exposed daily from 6 to 80 ng of anabasine (see Eq. 5).

4 $$DN{C}_{e}=SDN\times \frac{N{P}_{mean}}{Con{c}_{SUCROSE-nectar}}$$

5 $$Exp=DNC\times Con{c}_{Anab}$$

DNC_e_, daily nectar consumption estimate (μL); SDN, sucrose daily need (μg); NPmean, average nectar production (= 23.5 μL); Conc_SUCROSE-nectar_, sucrose concentration in *N. glauca* nectar; Exp, bees daily anabasine exposure (ng); Conc_Anab_, anabasine concentration in nectar (μg/mL).

## Discussion

The active ingredient of the feeding solution was the flower extract of *N. glauca*. The reason behind this choice over the nectar extract is related to the foraging habits of the honeybees on *N. glauca* flowers. In specific as clearly stated by Ollerton et al. [17] nectar robbers pierce the corolla, or make use of previous holes, and honeybees are totally capable to cut a hole in the corolla with their mandibles. The 10 μg/mL concentration was used as a focal point of our study. Such concentration was also assessed in feeding responses as presented by Singaravelan et al. (in artificial nectar) [30], and is approximately double the mean concentration in nectar reported by Tadmor-Melamed et al. [16].

It must be noted that honeybees’ foraging in *N. glauca* requires the consumption of herbal tissue to reach the flower’s nectar, as evidenced by our group and depicted by Ollerton et al. [17]. It is also noteworthy that damaging of petals-flowers by honeybees was evidenced (see also a respective photo taken during bees visiting *N. glauca* in Greece, Fig. 1), a fact known to elicit the alkaloidal response in *Nicotiana* spp. manifesting increase in concentrations in flowers and nectar. In addition, and as mentioned by Adler and colleagues, leaf alkaloid levels in *Nicotiana tabacum* are higher than the nectar alkaloid levels (mg/g compared to μg/g, respectively) [41], which can be hypothesized for *N. glauca* accordingly. Research findings concerning alkaloid levels in other species, such as lupin (*Lupinus* L. spp.), demonstrated higher levels of alkaloids in inflorescences, with respect to leaves, and stems [42]. Similarly, Gosselin et al. reported that alkaloids of toxic plant *Aconitum septentrionale* (Ranunculaceae) display lower concentrations in nectar compared to leaves and flowers. Hence, it cannot be excluded that bees can potentially be exposed to higher alkaloids’ concentrations than the ones present in non-damaged flowers or even nectar, which to a degree, can justify the 10 μg/mL concentration used in the assay. Such approach can also compromise the absence of nectar in testing and bridge the difference in estimated exposure and exposure dose(s) administered.

Interestingly, Pashalidou and colleagues reported that bumblebees in the scarcity of pollen, damage plant leaves stimulating flower production [43]. In this regard, it can be assumed that if bees experience respective scarcity in *N. glauca*, can further damage its leaves and flowers, which might lead to augmented alkaloids concentration. Nevertheless, it was not the case in this work due to the time of the sampling (*N. gla*u*ca* exhibits substantial blooming within August).

The increased mortality provoked by the administration of the methanol extract at 20 μg/mL, indicates that this observation might be connected to the presence of anabasine, which is a known insect control agent [44]. The potential association of anabasine with bees’ mortality should be treated with caution, considering that the administered plant extracts may contain other plant toxins, not included in the analytical method. Nevertheless, studies on *N. glauca* content have demonstrated several constituents such as sesquiterpenes, diterpenoids, and phenols in addition to alkaloids [45, 46]. In this context, such compounds although prevalent, are not expected to provoke substantial effects on bees, not omitting of course the combined effects that are to a large extent underexplored. For example, recent work on the beneficial role of such components showed that the terpenes of various parts of thyme inhibited the growth of bee disease associated microbes [47].

Singaravelan et al. [30] investigating the effects of free-flying bees’ exposure to secondary compounds that mimic floral nectars reported that anabasine was shown to deter honeybees, with the more profound effect being observed at the 25 μg/mL dose. In an ensuing study though delineating the nicotine impacts on honeybees, it was stressed that symptoms and mortality need to be examined in the context of the overall hygiene and bee health status [48]. Even though previous research results provide advocacy for present findings, are not directly comparable regarding the parameters examined herein, dictating that the role of anabasine in bees mortality needs to be investigated further through experimentation detailing the dose–response phenomena in a prolonged time frame. Nevertheless, present findings report for the first time the alkaloid content of *N. glauca* flowers, documenting them as a prominent source of invasive molecules in organisms foraging its flowers.

To estimate the bee’s daily alkaloid consumption was considered the maximum daily nectar consumption of 128 mg/bee for forager honeybees [49] and the density of the 50% w/v sucrose aqueous solution used (1.23 g/mL, 20 °C). Based on these assumptions the dose of anabasine in which a single honeybee was exposed within the 24 h-feeding period is determined at 1.04 μg/bee in feeding solution concentration of 10 μg/mL, and at 2.08 μg/bee in feeding solution concentration of 20 μg/mL. Minor discrepancies in the detected residues, among the three feeding solutions, might indicate differential behaviour of bees as regard the plant extract uptake during experimentation and/or differential metabolism within individual bees. The differences in mean measured concentrations in bees compared to the external doses are expected, considering that the internal dose is dependent on several factors. Among them, the most pivotal is the toxicant’s half-life (t_1/2_.), also mentioned in the European Food Safety Authority (EFSA) guidance document on risk assessment on bees [49]. More specifically, once an animal stops to be exposed to the toxicant, the anticipated time for its virtual elimination from the animal’s body is five-times its t_1/2_ [49]. Anabasine shares similar t_1/2_ with nicotine (2–3 h in plasma) [50], which along with the sampling time and the low bioaccumulation potential of the polar anabasine (and related alkaloids) can justify the low concentrations detected. The attribution of the residual quantities in individual bees indicates the possibility of the potential transfer of these residues inside the beehive (see paragraph below).

Considering the sample preparation for alkaloids extraction from bees, it was based on two approaches: (a) a methanolic and/or ethyl acetate direct extraction of alkaloids, using only a reconstitution (for ethyl acetate) and a drying step prior to chemical analysis and, (b) a modified QuEChERS protocol. The consideration of the QuEChERS methodology was attempted considering its extensive use in pesticides and organic contaminants sample preparation (and consequent analysis), and the possible introduction of alkaloids in such multiresidue schemes (nicotine possesses insecticidal properties). Specialized methods for alkaloids extraction exist, taking advantage of the acidobasic features and differences of various alkaloids solubility. Nevertheless, they were not implemented due to the more tedious sample preparation. Other laboratory trials (data not shown) on the optimization of QuEChERS protocol were attempted, especially on the pH adjustment (basic using NH_3_ or NaOH, pH ~ 9 to 10 on the first step, and addition of formic acid solution to reach a pH ~ 5 at the last step). Since the results were comparable to the sodium acetate standard procedure, we selected the latter as the final choice. In the end, both approaches (direct organic solvent and QuEChERS extraction) were efficient on recovering the targeted compounds as demonstrated by analytical method performance, with QuEChERS protocol, yielding cleaner extracts, therefore it was selected and fully validated. Alkaloids have been adequately extracted using QuEChERS from tea [51], tobacco [52], feed [53] and honey [54]. Therefore, this work contributes to the frontier of alkaloids extraction from another matrix (honeybees).

With regard to the hydrophilic stationary phase, it encourages partition of the analytes (in this case, the alkaloids and scopoletin) in the stationary water phase that is formed within the column. In addition, the preliminary comparison of RP with HILIC mode showed that the latter exhibited higher sensitivity for all analytes, possibly due to easier desolvation of the mobile phase (contained less water than the respective of RP) during the electrospray ionization.

**Fig. 3:**
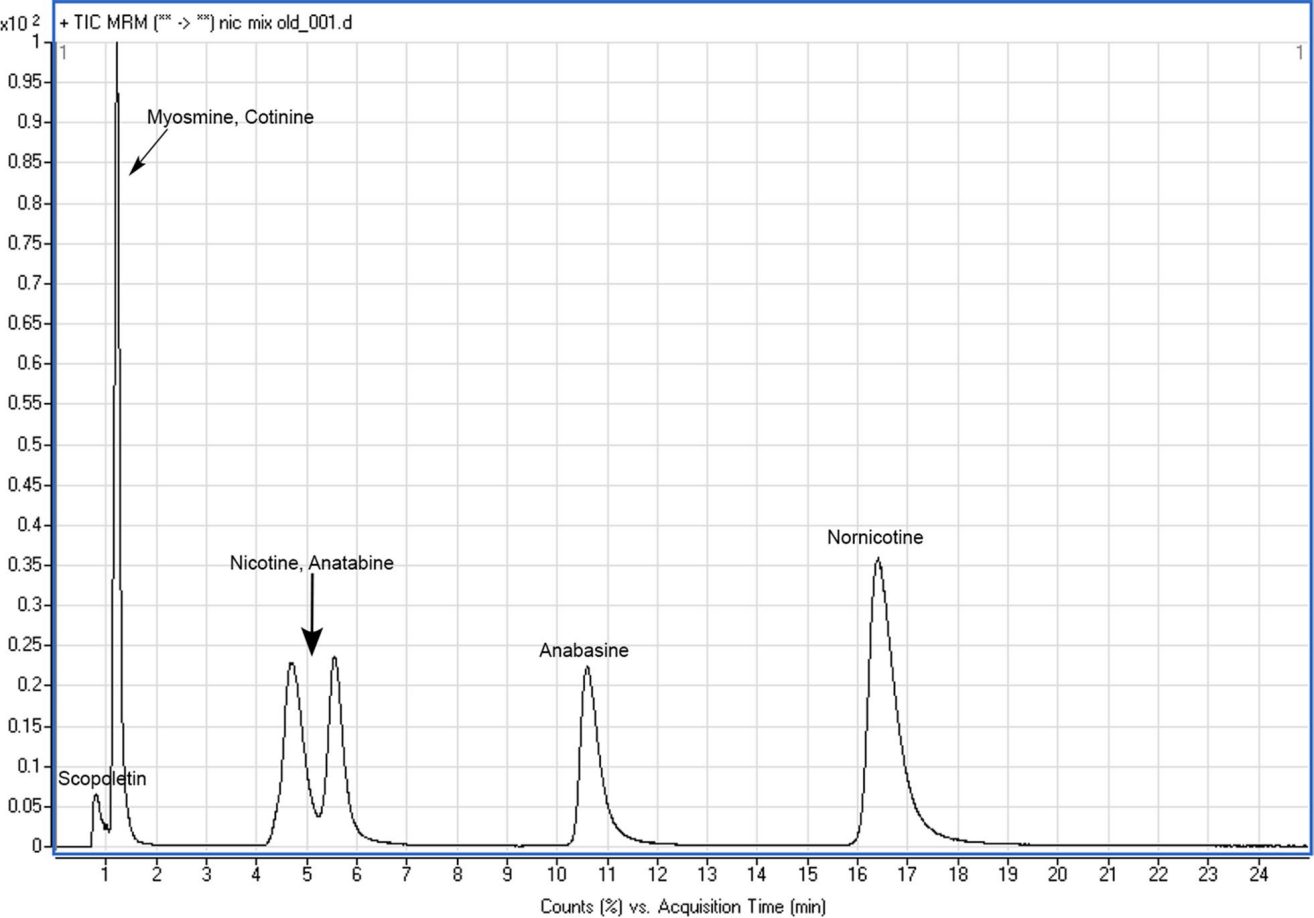
HILIC-ESI–MS/MS chromatogram of a 0.5 ppm standard solution of alkaloids mix

In this context, HILIC was implemented. The latter additionally exhibited superior performance compared to RP, C18 chromatography (for C18 chromatography see chromatogram in Additional file 1: Figure S6), improving the separation between the isomers especially for ( ±)-nicotine and (*R,S*)-anatabine, furnishing better chromatographic peak shapes for the majority of analytes, which is depicted in Fig. 3. With regard to the mobile phase selection, the trademark of low water content under HILIC conditions was verified in this study as well. Compared to previous work [31], the elution order of common analytes was verified, however in the present work the inclusion of more analytes, that in the HILIC mode were expected to elute early (such as scopoletin and myosmine), led to a decrease of the flow rate, selection of a column with larger film thickness; hence, enhancing the separation, though increasing the retention. Apart from the work of Taujenis and coworkers, a 2006 report showed also how the HILIC conditions employing low water amounts, favor the resolution and chromatographic performance of other alkaloids based on xanthine [55]. In previous studies, HILIC mode conditions were reported successful in separating low resolution isomers of dansyl amino acids and nicotine enantiomers analogs [56, 57]. A recent LC coupled to high resolution mass spectrometry (HRMS) research work also demonstrated the importance and optimum performance of HILIC in the separation of nortropane alkaloids (calystegines) in tomato-based products [58]. A clear advantage of this analytical work (and related works on alkaloids separation using HILIC) seems to be the distinct separation of anabasine and nicotine, whose structural-chromatographic differentiation is still a challenging topic [59].

To conclude, acknowledging the expansion of tobacco tree in the Northern Mediterranean coast, bees were placed in the center of this study, since along with other wild insects have been recorded feeding on its plentiful nectar. Therefore, the fundamental question was raised: do tobacco alkaloids, which have been defined as potent insecticides, penetrate in the foraging organisms? To answer this question, a novel method was developed to be able to quantify the tobacco tree alkaloids in the flower extracts and bees, and then a feeding bioassay in bees was performed. The proposed analytical methodology succeeded to detail the alkaloid content of the extracts and to provide qualitative and quantitative definition of the alkaloids residues in bees. The feeding bioassay provided indications of low to medium toxicity of the extracts but more importantly defined the alkaloid content of the dead bees, though in nature consumption by other organisms, is sparingly documented, focused to bee-eater birds (Coraciiformes: Meropidae) [60]. The impacts of *Nicotiana* alkaloids in honeybees were explicitly summarized by Stevenson et al. (2017) [61], identifying foraging repellent and attractive impacts depending on concentration a null impact on the survival of adult bees, which agrees with present findings, but also a reduced larvae survival rate both in vivo and in vitro. On the other hand, it a chemoprotective impact on the reduction in *Crithidia bombi* infection was also reported [62]. These facts in cross consideration with present findings advocate on the role and significance of the reported phenomenon on honeybee’s population dynamics. After all, Neov et al. (2019) [63] argued in favor of the role of feeding disorder in the beehives health status, also recognizing the significant impacts of neonicotinoids, agrochemicals defined as pyridine alkaloids. The role and significance of invasive alien species as honeybees feed sources has been stressed by Donkersley et al. [64] that identified a primary pollen source in the form of the invasive *Himalayan balsam*, providing a previous report that advocates the primary role of *Nicotiana glauca* as a honeybee feed source in arid Mediterranean habitats, especially through seasons with scarce flowering. On the contrary, the most important aspect not to be neglected is the transfer of such compounds in the beehive by forager honeybees and potentially to apiculture products, exemplified by honey. Such transfer though remains to be proven. Alkaloids in honey, such as pyrrolizidine based molecules (e.g., echimidine), are intensively studied in terms of residual prevalence [65, 66] and some of the members display carcinogenic, hepatotoxic and other toxic pharmacological activities [67]. Despite their toxicity, the European Commission has not yet established their MRLs.

Similarly, and with regard to pyridine alkaloids, such as nicotine and anabasine, none specific MRL is defined in honey. Therefore the general default MRL of 0.01 mg/kg can be regarded. Consequently, from the results of the presented study and the detection of anabasine in bees it can be assumed that the beehive might get contaminated from these alkaloids, and residues can be transferred to the honey produce and the food chain. Nevertheless, EFSA states that MRL of nicotine in different commodities ranging from 0.3–4 mg/kg, is not risk for consumers [68].

## Supplementary information

**Additional file 1. Figure S1.** TIC LC–ESI–MS/MS chromatogram of blank bees’ QuEChERS extract. **Figure S2.** Poor resolution of ( ±)-nicotine and (*R,S*)-anatabine under reversed phase chromatographic conditions^a^. **Figure S3.** A-G Retention times and MRM ion transitions (m/z) used in analysis by HILIC chromatography. **Figure S4.** Overlayed TIC chromatogram, and MRM chromatogram for anabasine in a Nicotiana glauca MeOH extract (100 ppm)^a^. **Figure S5.** TIC chromatogram (and MRM chromatogram of quantitation transition of anabasine) in bees. **Figure S6.** TIC LC–ESI–MS/MS chromatogram of a standard solution at 2 ppm of analytical standards mix using classical C18 column, and indicative MRM chromatograms (scopoletin, anatabine, myosmine and cotinine). **Table S1.** Bioactive components concentrations (μg/mL) in administered *Nicotiana glauca* extracts. **Table S2.** Analytical Method Validation Characteristics for the *Nicotiana glauca* hexane extract. **Table S3.** Analytical Method Validation Characteristics for the *Nicotiana glauca* dichloromethane extract. **Table S4.** Analytical Method Validation Characteristics in honeybees using the QuEChERS protocol.

## Data Availability

All necessary data and materials are presented in the manuscript and in the supplementary material.

## Abbreviations

Conc_Anab_: Anabasine concentration in nectar
NPmean: Average nectar production
Exp: Bees daily anabasine exposure
DNC_e_: Daily nectar consumption estimate
LOD: Detection limit
EFSA: European Food Safety Authority
HILIC: Hydrophilic interaction chromatography
IAS: Invasive Alien Species
MgSO_4_: Magnesium sulphate
ME: Matrix effect
LD_50_: Median lethal dose
LC–ESI–MS/MS: Liquid chromatography electrospray tandem mass spectrometry
MRLs: Maximum residue limits
MRM: Multiple Reaction Monitoring
C18: Octyldecylsilane
OECD: Organization for Economic Co-operation and Development
PSA: Primary secondary amine
LOQ: Quantitation limit
QuEChERS: “Quick, easy, cheap, effective, rugged, and safe”
RSD: Relative standard deviation
RP: Reversed phase
Conc_SUCROSE-nectar_: Sucrose concentration in *N. glauca* nectar
SDN: Sucrose daily need
TIC: Total ion chromatogram
