# Supplementary material for: An essay on ecosystem availability of Nicotiana glauca graham alkaloids: the honeybees case study
Source: BMC Ecol. 2020 Nov 6;20:57. doi: 10.1186/s12898-020-00325-3 (PMC7646078; doi:10.1186/s12898-020-00325-3)
Supplement: Supplementary file 1 — Additional file 1. Figure S1. TIC LC–ESI–MS/MS chromatogram of blank bees’ QuEChERS extract. Figure S2. Poor resolution of ( ±)-nicotine and (R,S)-anatabine under reversed phase chromatographic conditionsa. Figure S3. A-G Retention times and MRM ion transitions (m/z) used in analysis by HILIC chromatography. Figure S4. Overlayed TIC chromatogram, and MRM chromatogram for anabasine in a Nicotiana glauca MeOH extract (100 ppm)a. Figure S5. TIC chromatogram (and MRM chromatogram of quantitation transition of anabasine) in bees. Figure S6. TIC LC–ESI–MS/MS chromatogram of a standard solution at 2 ppm of analytical standards mix using classical C18 column, and indicative MRM chromatograms (scopoletin, anatabine, myosmine and cotinine). Table S1. Bioactive components concentrations (μg/mL) in administered Nicotiana glauca extracts. Table S2. Analytical Method Validation Characteristics for the Nicotiana glauca hexane extract. Table S3. Analytical Method Validation Characteristics for the Nicotiana glauca dichloromethane extract. Table S4. Analytical Method Validation Characteristics in honeybees using the QuEChERS protocol. [file 12898_2020_325_MOESM1_ESM.docx]

**Supplementary material**

INVASIVE MOLECULES: AN ESSAY ON ECOSYSTEM AVAILABILITY AND PENETRABILITY OF *Nicotiana glauca* GRAHAM ALKALOIDS

KONSTANTINOS M. KASIOTIS,^1,*^ EPAMEINONDAS EVERGETIS,^2,*^ DIMITRIOS PAPACHRISTOS,^3^ OLYMPIA VANGELATOU,^2^ SPYRIDON ANTONATOS,^3^ PANAGIOTIS MILONAS,^4^

SERKOS A. HAROUTOUNIAN^2^ and KYRIAKI MACHERA^1^

*^1^Benaki Phytopathological Institute, Department of Pesticides Control and Phytopharmacy, Laboratory of Pesticides’ Toxicology, 8 St. Delta Street, Kifissia, 14561 Attica, Greece*

*^2^Laboratory of Nutritional Physiology and Feeding, Agricultural University of Athens, Iera Odos 75, 11855 Athens, Greece*

*^3^ Benaki Phytopathological Institute Department of Entomology and Agricultural Zoology, Laboratory of Agricultural Entomology, 8 St. Delta str., Kifissia, 14561 Attica, Greece;*

*^4^ Benaki Phytopathological Institute Department of Entomology and Agricultural Zoology, Biological Control Laboratory, 8 St. Delta str., Kifissia, 14561 Attica, Greece*

*Corresponding authors:

Konstantinos M. Kasiotis, Laboratory of Pesticides’ Toxicology, Department of Pesticides Control and Phytopharmacy, Benaki Phytopathological Institute, 8 St. Delta Str., Kifissia 14561, Athens, Greece. Tel: 00302108180357, Email: [K.Kasiotis@bpi.gr](mailto:K.Kasiotis@bpi.gr)

Epameinondas Evergetis: Laboratory of Nutritional Physiology and Feeding, Agricultural University of Athens, Iera Odos 75, 11855 Athens, Greece. Tel: +302105294246, Email: [epaev@mac.com](mailto:epaev@mac.com)

**Figures**

**FIG. S1** TIC LC-ESI-MS/MS chromatogram of blank bees’ QuEChERS extract

|  |
| --- |

**FIG. S2** Poor resolution of (±)-nicotine and (R,S)-anatabine under reversed phase chromatographic conditions^a^

|  |
| --- |

^a^red line corresponds to (R,S)-anatabine TIC chromatogram

**FIG. S3A-G** Retention times and MRM ion transitions (m/z) used in analysis by HILIC chromatography

| **A. Anabasine** | **B. Scopoletin** | **C. Nicotine** |
| --- | --- | --- |
|  |  |  |
| **D. Cotinine** | **E. Nornicotine** | **F. Myosmine** |
|  |  |  |
| **G. Anatabine** | | |
|  | | |

**FIG. S4** Overlayed TIC chromatogram, and MRM chromatogram for anabasine in a Nicotiana glauca MeOH extract (100 ppm)^a^

|  |
| --- |

^a^green line corresponds to 163 → 117 MRM quantitation transition

**FIG. S5** TIC chromatogram (and MRM chromatogram of quantitation transition of anabasine) in bees

**FIG. S6** TIC LC-ESI-MS/MS chromatogram of a standard solution at 2 ppm of analytical standards mix using classical C18 column, and indicative MRM chromatograms (scopoletin, anatabine, myosmine and cotinine)

| 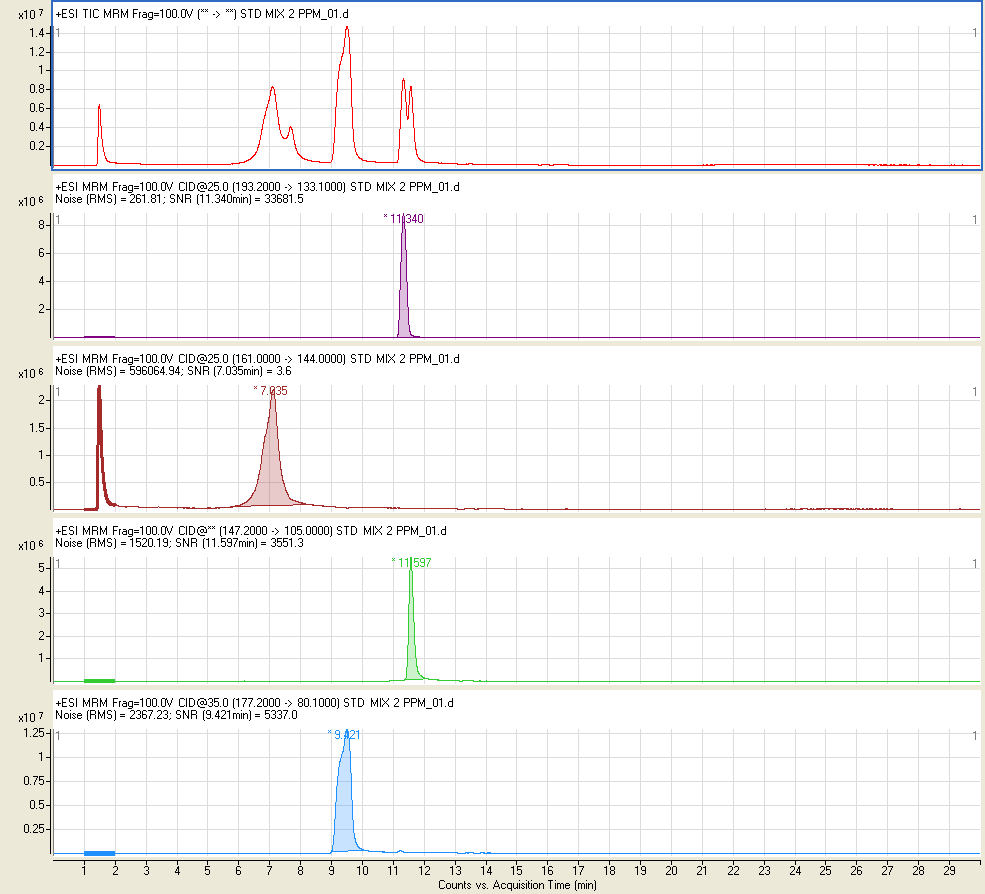 |
| --- |

**Tables**

**Table S1:** Bioactive components concentrations (μg/mL) in administered *Nicotiana glauca extracts*.

| **Constituent** | ***Nic. glauca MeoH*** | ***Nic. glauca DCM*** | ***Nic. glauca Hex*** |  |
| --- | --- | --- | --- | --- |
| Anabasine | 10 | 10 | 10 |  |
| Anatabine | 0.097 | 0.115 | nd |  |
| Scopoletin | 0.105 | nd | nd |  |
| Nornicotine | 0.03 | nd | nd |  |

nd: non-detected

**Table S2.** Analytical Method Validation Characteristics for the *Nicotiana glauca* hexane extract**.**

| **Analyte** | **Regression Equation*** | **Regression coefficient (R^2^)** | **ME (%)** | **Recovery ±RSD % (n = 3)** | | | **Inter-d precision**  **(RSD % n = 3)** | **Intra-d-precision**  **(RSD % n = 3)** |
| --- | --- | --- | --- | --- | --- | --- | --- | --- |
|  |  |  |  | 50 ng/g | 200 ng/g | 1000 ng/g | 200 ng/g | 200 ng/g |
| Anabasine | y=6300160x-432908,1 | 0.9981 | -1.6 | 86±10 | 90±12 | 86±7 | 8.2 | 6.3 |
| Nicotine | y=1241081x+10098 | 0.9990 | 7.7 | 77±8 | 79±7 | 89±9 | 4.9 | 3.9 |
| Anatabine | y=4435921+4430 | 0.9995 | 7.2 | 90±12 | 84±10 | 81±8 | 6.2 | 5.5 |
| Nornicotine | y=61042304,9x-401500,2 | 0.9987 | -6.0 | 93±11 | 93±16 | 85±7 | 11.2 | 8.7 |
| Myosmine | y=5630098,1x-22985 | 0.9998 | 9.1 | 77±8 | 76±9 | 80±5 | 9.9 | 10.8 |
| Scopoletin | y=1192039,7x-1980,5 | 0.9990 | -2.7 | 82±5 | 82±8 | 92±6 | 3.9 | 4.3 |
| Cotinine | y=27830942x-110447 | 0.9983 | 5.6 | 83±7 | 82±14 | 91±9 | 5.8 | 5.0 |

*Residuals for all concentration levels were below 16%.

**Table S3.** Analytical Method Validation Characteristics for the *Nicotiana glauca* dichloromethane extract**.**

| **Analyte** | **Regression Equation*** | **Regression coefficient (R^2^)** | **ME (%)** | **Recovery ±RSD % (n = 3)** | | | **Inter-d precision**  **(RSD % n = 3)** | **Intra-d-precision**  **(RSD % n = 3)** |
| --- | --- | --- | --- | --- | --- | --- | --- | --- |
|  |  |  |  | 50 ng/g | 200 ng/g | 1000 ng/g | 200 ng/g | 200 ng/g |
| Anabasine | y=6000150x-32345,8 | 0.9986 | -4.8 | 82±9 | 88±10 | 87±7 | 10.1 | 3.8 |
| Nicotine | y=1201899x+10061 | 0.9994 | 6.9 | 78±12 | 81±11 | 87±8 | 3.9 | 5.2 |
| Anatabine | y=5435018+5670.9 | 0.9990 | 8.1 | 100±15 | 85±10 | 79±11 | 5.0 | 4.2 |
| Nornicotine | y=62048560x-312209 | 0.9989 | -5.8 | 91±14 | 90±15 | 85±9 | 8.3 | 5.3 |
| Myosmine | y=4902432,7x-27980 | 0.9990 | 9.0 | 81±10 | 80±4 | 80±9 | 11.2 | 8.1 |
| Scopoletin | y=1588939x-4092,8 | 0.9990 | -2.8 | 78±6 | 83±5 | 90±8 | 7.7 | 6.3 |

*Residuals for all concentration levels were below 15%.

**Table S4.** Analytical Method Validation Characteristics in honeybees using the QuEChERS protocol

| **Analyte** | **Regression Equation*** | **Regression coefficient (R^2^)** | **ME (%)** | **Recovery ±RSD % (n = 3)** | | | **Inter-d precision**  **(RSD % n = 3)** | **Intra-d-precision**  **(RSD % n = 3)** |
| --- | --- | --- | --- | --- | --- | --- | --- | --- |
|  |  |  |  | 40 ng/g | 200 ng/g | 1000 ng/g | 200 ng/g | 200 ng/g |
| Anabasine | y=6000049,8x-30350,2 | 0.9982 | -3.8 | 86±11 | 87±10 | 83±8 | 7.6 | 2.8 |
| Nicotine | y=1201011x+8890,3 | 0.9994 | 5.8 | 78±12 | 81±11 | 87±8 | 3.9 | 5.2 |
| Anatabine | y=3935018+1908,9 | 0.9990 | 7.2 | 100±15 | 85±10 | 79±11 | 5.0 | 4.2 |
| Nornicotine | y=58048304x-401000,5 | 0.9982 | -5.8 | 91±14 | 90±15 | 85±9 | 8.3 | 5.3 |
| Myosmine | y=4087432,2x-25608,1 | 0.9990 | 8.4 | 75±5 | 75±4 | 80±14 | 8.1 | 10.5 |
| Scopoletin | y=1099939,4x-2709,4 | 0.9995 | -0.8 | 74±11 | 82±5 | 94±8 | 3.1 | 7.2 |

*Residuals for all concentration levels were below 15%.
